# Design and Experimental Investigation of an Ultra-Low Frequency, Low-Intensity, and Multidirectional Piezoelectric Energy Harvester with Liquid as the Energy-Capture Medium

**DOI:** 10.3390/mi14020369

**Published:** 2023-02-01

**Authors:** Ning Li, Fan Yang, Tao Luo, Lifeng Qin

**Affiliations:** 1 Shenzhen Research Institute of Xiamen University, Shenzhen 518000, China; 2 Department of Mechanical and Electrical Engineering, School of Aerospace Engineering, Xiamen University, Xiamen 361005, China

**Keywords:** piezoelectric vibration energy harvester, low frequency, low intensity, multidirectional

## Abstract

Traditional piezoelectric vibration energy harvesters (PVEHs) usually adopt a rigid energy-capture structure, which can achieve efficient energy harvesting in single-directional, high-frequency, and high-intensity vibration environments. However, efficient harvesting with the use of low-frequency, low-intensity, and multidirectional vibration energy remains a challenge for existing harvesters. To tackle this problem, we proposed a PVEH with liquid as the energy-capture medium. Our previous research verified that this set up can show a good energy harvesting performance under low-frequency, low-intensity, and horizontal multidirectional vibration excitation. In this paper, we further studied the possibility of vertical multidirectional energy harvesting using this device, as well as the influence of several important parameters (rope margin, liquid level height, and floating block shape) on the output performance. The results showed that the proposed PVEH can realize energy harvesting in three-dimensional space and that the output characteristic is adjustable.

## 1. Introduction

With the rapid development of wireless electronic devices and communication technology, wireless sensor networks and the Internet of Things are becoming more widely used. In addition, batteries, as traditional energy supply devices, are also facing the challenges of limited service life and large-scale replacement. These problems are expected to be solved as more energy harvesters capable of harvesting energy from triboelectricity [1], solar radiation [2], heat [3], or vibration [4] are being widely studied. Piezoelectric vibration energy harvesters (PVEHs) have attracted increasing attention from researchers due to their applicability and high power density [5,6,7,8].

Traditional PVEHs usually consist of a high-frequency piezoelectric cantilever and mass block; the energy-capture medium is a rigid material that can harvest energy effectively in a specific direction at high frequencies [9]. In addition, for the harvesting of low-frequency, low-intensity, and multidirectional energy [10,11,12] by PVEHS, a frequency up-conversion mechanism and a multi-piezoelectric beam structure are often introduced. Liu et al. [13] developed an energy harvester containing a double-beam structure, which first vibrated the low-frequency beam under the action of low-frequency excitation in the direction of the beam’s thickness and achieved frequency up-conversion through the collision between beams vibrating at both low and high frequencies to achieve low-frequency energy harvesting. Wang et al. [14] developed a cross-coupled dual-beam structure that comprised two sets of three beams joined crosswise and a circular cylinder bluff-body attached to the free end of the bottom beam, which reduced the resonance frequency of the device to 12.23 Hz and enabled the device to harvest multidirectional wind energy in a two-dimensional (2D) plane. Zhang et al. [15] proposed a tridirectional piezoelectric energy harvester composed of the main beam, a rolling ball, and a hemispherical shell, and it achieved a normalized power density of 22.63 µW/(cm^3^g^2^Hz) under the action of 3D multidirectional external excitation at a low frequency (7.6 Hz) and low intensity (0.03 g).

However, efficient energy harvesting under the excitation of numerous vibration sources, including ultra-low frequency (<3 Hz), low-intensity (≤0.04 g), and multidirectional vibration sources in the surrounding environment, such as human movement and built-up vibration caused by strong winds [16,17,18,19], remains a challenge for the existing PVEHs [20,21,22]. Such limitations may be caused by the device’s energy-capture medium. Moreover, given the small intermolecular force, liquids have a good fluidity and low starting barrier, and PVEHs using liquids as an energy-capture medium have a better energy harvesting performance in ultra-low frequency (<3 Hz), low-intensity (≤0.04 g), and multidirectional (360° in the horizontal plane) vibration environments, as has been verified in our previous study [23]. In this paper, the multidirectional performance of a PVEH in the vertical plane and the influences of structural parameters (rope margin, liquid level height, and floating block shape) on its output performance were studied. The experimental results demonstrated that PVEHs using liquid as an energy capture medium has a high feasibility for ultra-low frequency (<3 Hz) and low intensity (≤0.04 g) energy harvesting in a 3D vibration environment, and its output performance can be promoted by optimizing the structural parameters.

This article is organized as follows. Section 2 introduces the system design and theoretical analysis of PVEHs. Section 3 discusses the construction of the PVEH prototype and the experimental test bench. Section 4 demonstrates the performance of energy harvesters in ultra-low frequency, low-intensity, and multidirectional vibration environments through experiments and practical applications. Section 5 provides the conclusions drawn from the study.

## 2. Working Principle of the Proposed PVEH

**Figure 1:**
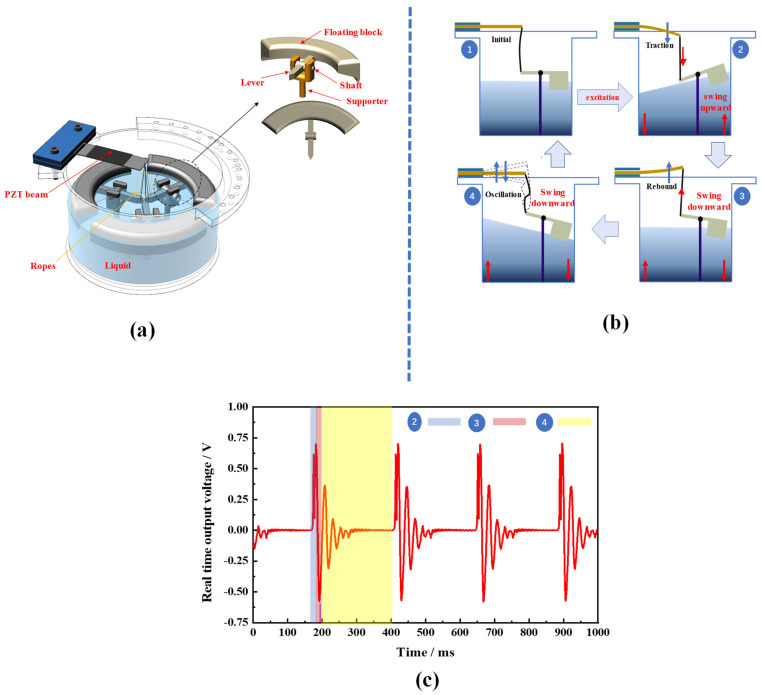
(**a**) Components of the PVEH; (**b**) operating principle of the PVEH; (**c**) real−time output voltage of the PVEH.

The proposed PVEH was composed of a cylindrical container holding a certain amount of liquid (water), a piezoelectric beam, floating blocks, and ropes connecting the floating blocks to the beam (Figure 1a). The piezoelectric beam was fixed at the upper end of the container by means of a splint. The floating blocks were constrained in a specific position by the support frame and the rotary shaft, and could only swing up and down around the rotary shaft. The vibration of the liquid caused the floating block to swing up and down around the rotary shaft, and when the swing amplitude became greater than the rope margin, the tightened rope will be pulled on the piezoelectric beam and deformed it. As shown in Figure 1b, the beam was pulled once in a working cycle, and it started oscillating. Electric energy was produced by the piezoelectric beam based on the piezoelectric effect. To gain a better understanding of the working principle of the proposed PVEH, we determined its real-time waveform (Figure 1c), which corresponded to different working states of the device.

**Figure 2:**
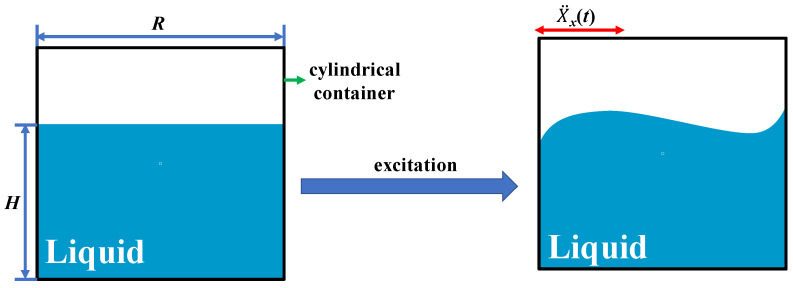
Schematic diagram of the liquid shaking.

The low-frequency characteristics of the device was briefly analyzed based on the shaking characteristics of the liquid in the cylindrical container. To simplify the analysis process, we used the effect of the floating blocks on the liquid’s vibration, which was ignored here, and horizontal excitation ($\ddot{X}$_x_*(t)*) as an external excitation (Figure 2). At this point, the first-mode resonance frequency (*f*) of liquid shaking can be calculated as follows ([24]):

(1) $$f=\sqrt{\frac{g\delta }{R}tanh(\frac{H\delta }{R})}/2\pi$$

where *H*(*m*) is the liquid height, *R*(*m*) is the inner wall radius of the cylindrical container, *g* = 9.8 m/s^2^ is the acceleration of gravity, and *δ* = 1.841 is the modal damping ratio coefficient for liquid shaking. According to Equation (1), the shaking frequency of the liquid in the container depends on the values of *R* and *H*, and when the container’s radius *R* is 50 mm and the liquid height *H* is 19 mm, the oscillation frequency f of the liquid is equal to approximately 2.35 Hz. In summary, by constantly adjusting the values of the container radius *R* and liquid height *H*, the device can achieve ultra-low frequency energy harvesting.

**Figure 3:**
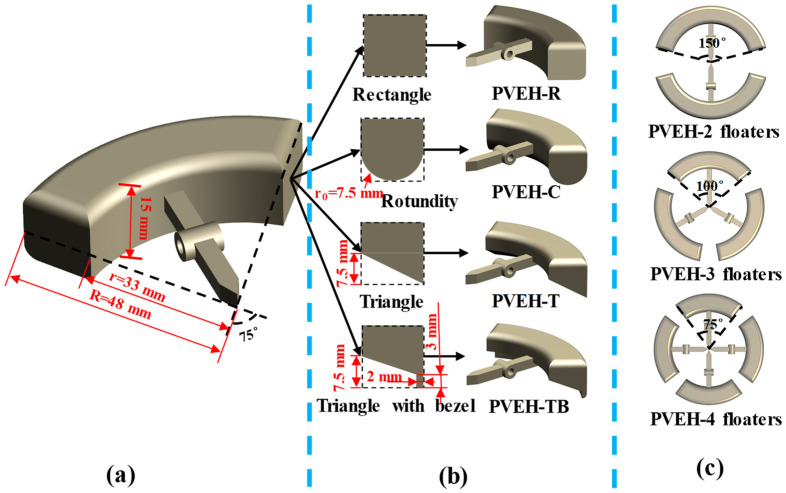
(**a**) Fan-shaped ring hollow floating block with lever; (**b**) floating blocks of different bottom shapes; (**c**) floating blocks with different array distributions.

The characteristics of multidirectional energy harvesting are achieved by arranging array cantilever beams inside conventional energy harvesters [25,26]. However, PVEHs with this structure require a complicated management circuit given the phase difference between each piezoelectric element [27]. In this paper, we achieved the multidirectional performance of the device by increasing the number of floating blocks, using one cantilever beam to achieve multidirectional energy harvesting. Three prototypes with different numbers of floating blocks were designed with the same total area of floating blocks (Figure 3c). They were equipped with two floaters with a 150° opening angle (PVEH-2 floaters), three floating blocks with a 100° opening angle (PVEH-3 floaters), and four floating blocks with a 75° opening angle (PVEH-4 floaters). The total coverage angle of the floating blocks in each prototype was 300°, and the remaining 60° range ensured that a certain gap existed between the floating blocks. Thus, no interference was observed between the floating blocks when swinging up and down.

**Figure 4:**
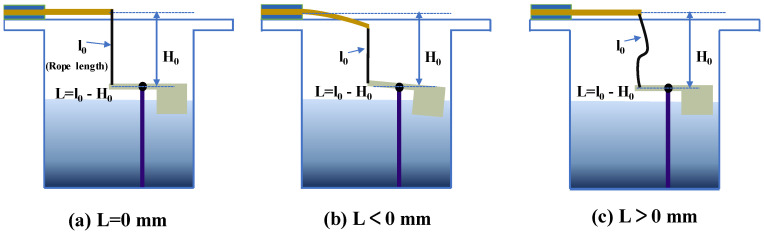
Schematic of the rope margin: (**a**) rope margin *L* = 0; (**b**) rope margin *L* < 0; (**c**) rope margin *L* > 0.

For the work process of the device, a coupling exists between the shaking liquid and floating block, and the shape of floating block will affect the interaction between the liquid and floating block and the output performance of the device. Therefore, four floating blocks with different bottom shapes were designed to study their effect on the output performance of the proposed PVEH. The four bottom shapes were rectangular (PVEH-R), circular (PVEH-C), triangular (PVEH-T), and triangular with a baffle (PVEH-TB) (Figure 3b). In addition, given that the rope was the medium of energy transfer between the floating block and cantilever beam, its degree of tension (rope margin *L*) affected the energy transfer efficiency (Figure 4), which was also studied in this paper.

## 3. Fabrication and Experiment Setup

**Figure 5:**
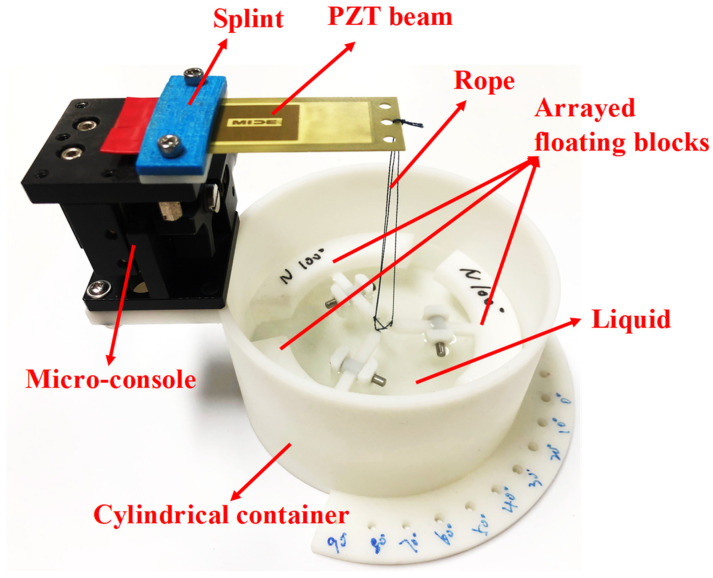
The prototype of the proposed PVEH.

A prototype was fabricated to examine the feasibility of PVEH for achieving vibrational energy harvesting in a low-frequency, low-intensity, and multidirectional excitation environment (Figure 5). The cylindrical container, whose inner diameter was 50 mm, and the floating block were produced via 3D printing using photosensitive resin as the material (SOMOS Imagine 8000, Royal DSM Group, The Netherlands). The floating blocks were designed as hollow structures to reduce their mass and shake them more easily. In addition, to generate multidirectional excitation in the device, we designed a lower platform on the container and fabricated several array-type round holes on the platform. By adjusting the position of these round holes, the direction of the excitation in the horizontal plane could be changed, and the exciter could change the direction of the excitation in the vertical plane. Thus, the direction of excitation acting on the device could be modified. Moreover, to study the effect of the rope margin on device performance, we fabricated an upper platform on the container, which was used to fix the micro-console, and the rope margin could be changed by adjusting the micro-console. The piezoelectric cantilever beam (PZT 5J S230-J1FR-1808XB) in the device was produced by Mide Technology Company in the United States, and the specific parameters are shown in Table 1.

**Figure 6:**
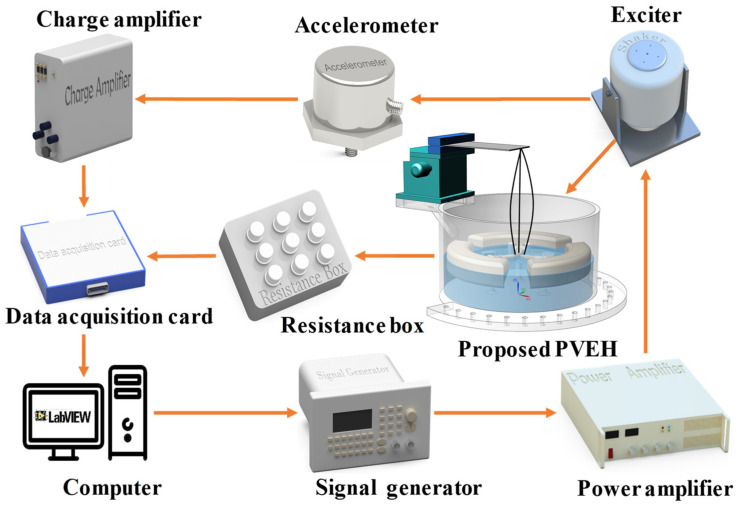
Experimental setup for the proposed PVEHs.

An experimental setup for PVEH was established (Figure 6). The prototype was mounted on the exciter via a fixture, and the vibration was provided by the exciter. Before that, the upper computer controlled the output of the signal generator through the program. The output port of the signal generator was connected to a power amplifier to achieve signal amplification, and the exciter receiving this signal began to vibrate. In addition, to maintain the stability of excitation, we observed records of the accelerometer mounted on the exciter table, the results showed acceleration data in real time and fed them back to the host computer. Moreover, as the external load of the device, the voltage signal of the resistance box was recorded and saved by the data acquisition card, and the output power of the device could be calculated by the recorded voltage.

## 4. Results and Discussion

**Figure 7:**
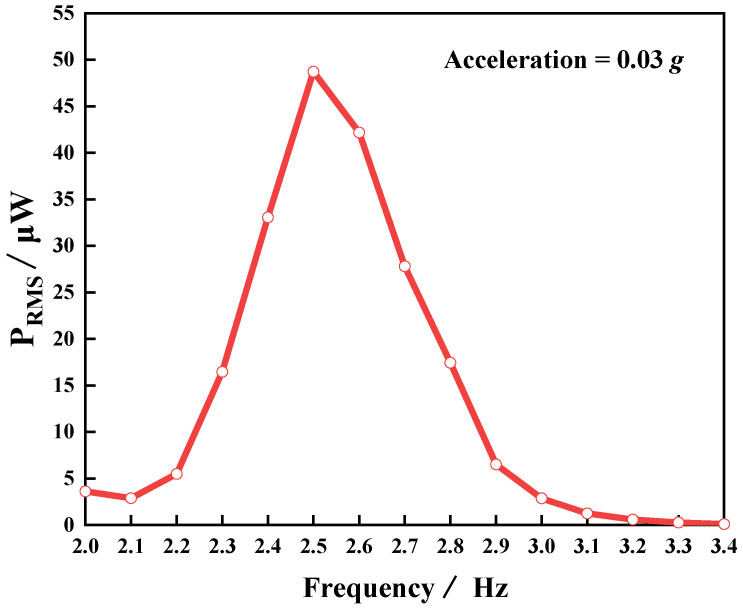
P_RMS_ of PVEH- R as a function of frequency.

To confirm the ultra-low frequency (≤3 Hz) and low-intensity (≤0.04 g) characteristics of the device, we used a PVEH-R device containing one floating block in the experiment (*H* = 19 mm, *L* = 0 mm). Figure 7 shows the root mean square (RMS) value of the device’s output power (P_RMS_) as a function of frequency at an acceleration of 0.03 g. Figure 7 also reveals that the resonant frequency of the device was as low as 2.5 Hz, which is very close to the 2.35 Hz calculated in Equation (1). The possible cause of the deviation was the effect of the floating block on the liquid shaking. In addition, with the change in external excitation frequency, the output power of the device reached a maximum of 48.71 µW at the resonant frequency. The above experimental results show that the device can achieve ultra-low frequency and low-intensity vibration energy harvesting.

**Figure 8:**
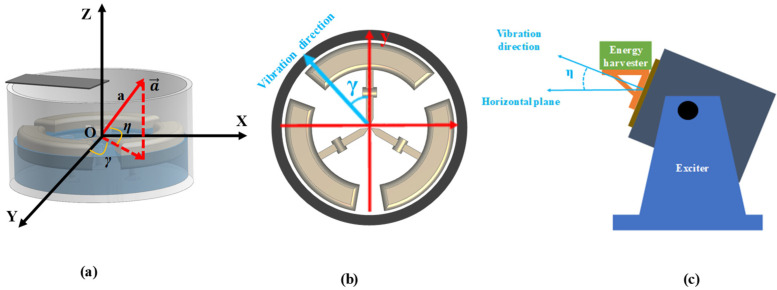
Excitation acceleration in ambient environment: (**a**) excitation acceleration in arbitrary directions; (**b**) acceleration in the horizontal plane; (**c**) acceleration in a vertical plane.

To study the multidirectional performance of the device in the 3D space, we established a spherical coordinate system to define the direction and value of the excitation intensity (acceleration $\vec{a}$). As shown in Figure 8a, the *X*-axis was established along the beam length, the *Y*-axis was established along the beam width, and the *Z*-axis was perpendicular to the XOY plane. Any acceleration in 3D space (Figure 8a) can be fully expressed in this coordinate system by the azimuth angle *γ*, elevation angle *η*, and acceleration value a. Therefore, the multidirectional performance of the device in 3D space (Figure 8b,c) can be studied separately in two planes: horizontal and vertical.

**Figure 9:**
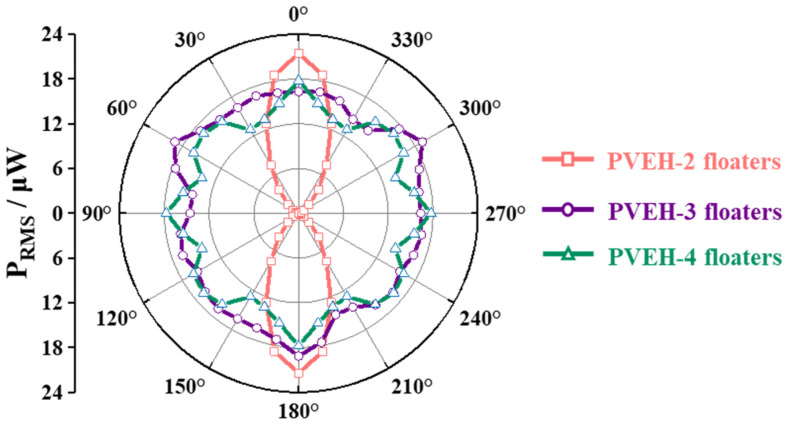
The angular response of PVEH with different numbers of floating blocks on the horizontal plane at ***a*** = 0.03 g, *H* = 23 mm, *L* = 0 mm.

**Figure 10:**
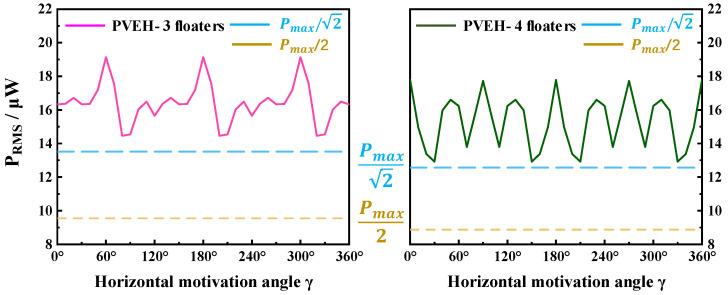
The horizontal angular bandwidth of PVEH-3 floaters and PVEH-4 floaters at their respective resonant frequencies.

In the horizontal plane, the multidirectional characteristics of PVEH can be achieved by increasing the number of floating blocks. As shown in Figure 8b, the direction of horizontal excitation can be adjusted by changing angle *γ*. Figure 9 reveals the angular response (symmetry of the effective output power of the device under vibration excitation in different directions) of each device in the horizontal plane. From Figure 9, PVEH-2 floaters exhibited a poor angular response at the resonant frequency (3.2 Hz), and the effective output power attenuation ((P_max_ − P_min_)/P_max_) in different directions reached 99%. However, in PVEH-3 (2.9 Hz) and PVEH-4 floaters, although the output characteristics of the device in the local range were reduced to a certain extent, the angular response was greatly improved. When the angular response of the device was evaluated by the angular bandwidth [26] (the sum of angles where the effective power value exceeds 1/2 P_max_) (Figure 10), the horizontal angular bandwidth of PVEH-3 and PVEH-4 floaters at the resonant frequency (2.7 Hz) was 360° (***a*** = 0.03 g, *H* = 23 mm; *L* = 0 mm). In addition, PVEH-3 and PVEH-4 floaters could still achieve a 360° angular bandwidth in the horizontal plane, despite the evaluation of the angular response of the device being at a high standard (√2/2 P_max_) (Figure 10). By reasonably selecting the number of floating blocks, the device can achieve all-around energy harvesting in the horizontal plane.

**Figure 11:**
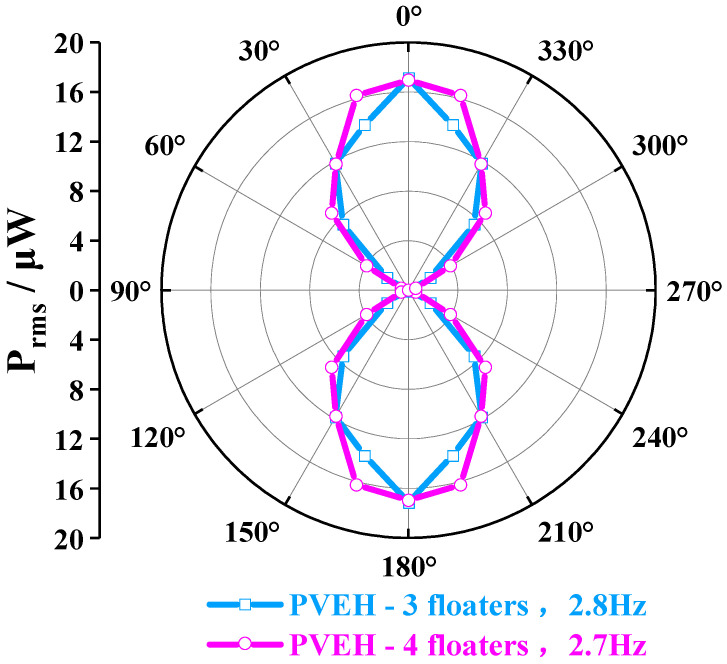
The angular response of PVEH-3 floaters and PVEH-4 floaters in a vertical plane.

In addition to horizontal multidirectional characteristics, the vertical multidirectional features of the device were studied; compared with numerous conventional energy harvesters that can only harvest energy in the 2D plane [28,29], the proposed PVEH in this paper can harvest energy in a vertical plane other than the horizontal plane due to the shaking characteristics of the liquid. As shown in Figure 8c, the angular response of the device in the vertical plane could be tested by adjusting the exciter to change the value of *η*. Under external conditions of ***a*** = 0.03 g, *H* = 23 mm, *L* = 0 mm, and *γ* = 0°, the PVEH-3 and PVEH-4 floaters could still obtain angular bandwidths of 120° in different excitation directions in the vertical plane (using √2/2 P_max_ as th standard) (Figure 11). Based on the experimental results mentioned above, the proposed PVEH can realize energy harvesting in multiple directions in 3D space under an ultra-low frequency and low-intensity excitation vibration.

**Figure 12:**
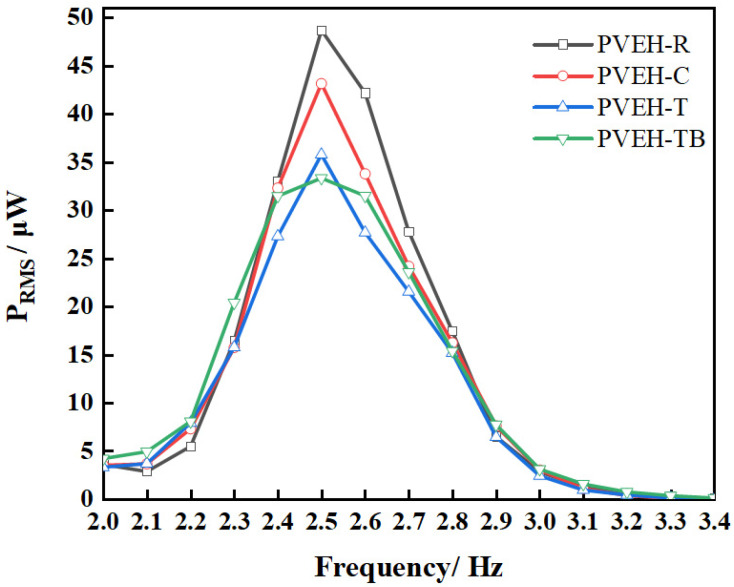
The effect of float shape on the PVEH output characteristics.

Figure 12 shows the effect of different float bottom shapes on the output characteristics of the device and the proposed PVEH with different floating blocks using the PVEH-1 floater as an example. The resonant frequency of PVEHs was 2.5 Hz when the intensity of the excitation was 0.03 g and *L* was 0 mm. The output powers of the different PVEHs were 48.71 (PVEH-R), 43.16 (PVEH-C), 35.79 (PVEH-T), and 33.35 µW (PVEH-TB). The different shapes of floating blocks resulted in various output performances, and the output power reached the maximum when the rectangle floating block was used. This finding may be due to the capability of the shape of the float bottom to change the coupling between the liquid and the float, thereby changing the energy-capture efficiency and energy transfer efficiency of the liquid. Thus, the output performance of the proposed PVEH can be improved by optimizing the float shape.

**Figure 13:**
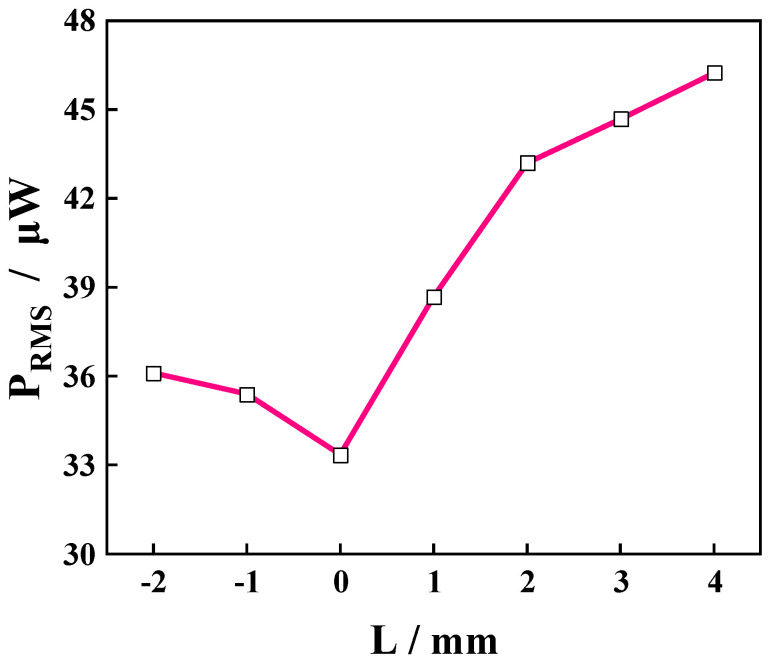
The effect of the rope margin on the PVEH output characteristics.

Figure 13 shows the effect of the rope margin on the output characteristics of the device taking the PVEH-TB floater as an example. When the rope margin was increased from −2 mm to 0 mm, the external output capacity of the device was constantly weakened, and the P_RMS_ dropped from 36 µW to 33 µW. However, as the rope margin *L* further widened from 0 mm to 4 mm, the device output increased, and P_RMS_ increased from 33 µW to around 46 µW. The influence of the rope margin on the output performance of the device was thus significant. The rope margin’s *L* affected the output characteristics of the device because it changed the time point at which the piezoelectric cantilever beam intervened in the coupling movement of the liquid and floating block, which modified the excitation of the piezoelectric cantilever beam. When the rope margin was *L* < 0 mm, the tension in the rope was greater than 0 in the initial state, the floating block was more submerged in the water due to the tension of the rope on it, and the piezoelectric cantilever beam was bent due to the reaction force of the rope. At this point, the piezoelectric cantilever beam intervened in the coupling movement between the liquid and float before excitation was applied. When *L* ≥ 0 mm, the tension of the rope was 0, the piezoelectric cantilever beam only intervened in the coupling movement of the liquid and floating block when it moved to or above the equilibrium position, and the time point of intervention was determined by the rope margin *L*. Therefore, by adjusting the margin of the rope reasonably, the output performance of the device can be improved.

**Figure 14:**
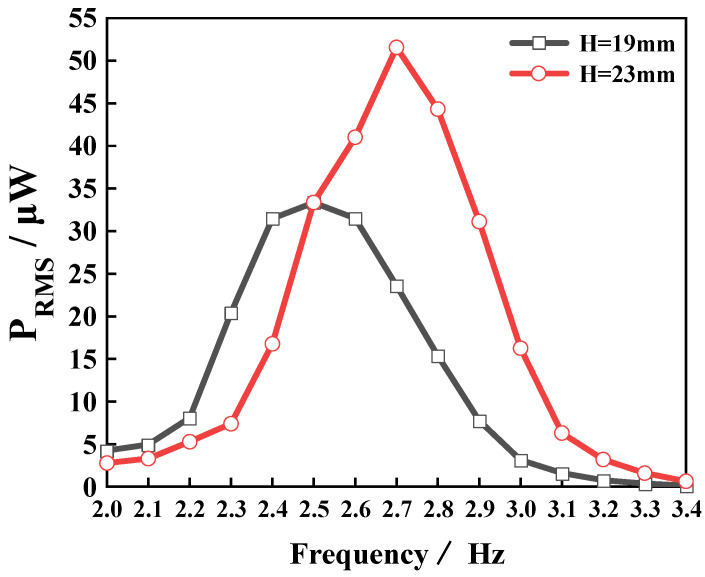
The effect of the liquid height on the PVEH output characteristics.

Figure 14 shows the resonant frequency and output performance of the device as a function of the liquid height. When the liquid height *H* increased from 19 mm to 23 mm, the resonant frequency of the device changed from 2.5 Hz to 2.7 Hz, and the effective output power P_RMS_ at the resonant frequency increased from 33.35 µW to 51.55 µW. This finding can be explained by Equation (1), that is, as *H* increases, the resonant frequency of the device also increases. Thus, the device harvested more energy in the external environment at the resonant frequency, and the external manifestation was the increased device output. This finding further confirmed the feasibility of adjusting the resonant frequency and output characteristics of the device by varying the liquid height. In summary, various device parameters have a significant influence on the device performance. Thus, to improve the device output performance, the relationship between various device parameters should be reasonably adjusted and coordinated.

**Figure 15:**
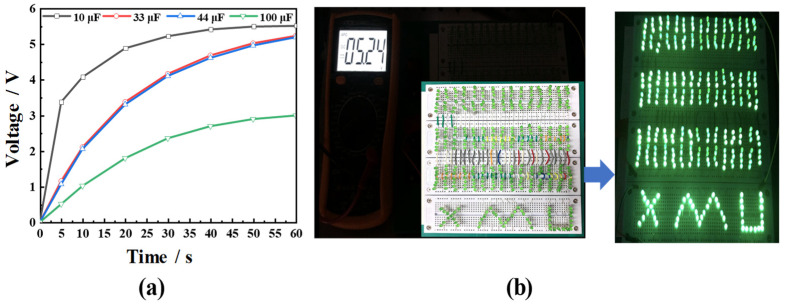
Demonstration of the proposed PVEH powering a capacitor: (**a**) the charging effect of different capacitors in the same time; (**b**) 333 LEDs lit in parallel.

**Figure 16:**
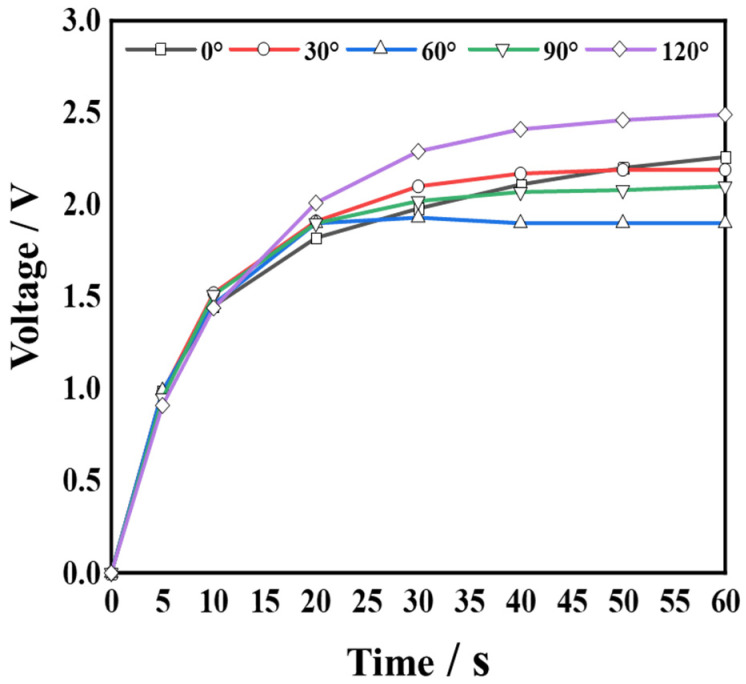
The effect of charging the capacitor for 60 s at a frequency of 2.7 Hz, ***a*** = 0.03 g, *H* = 23 mm.

Finally, the fabricated PVEH was connected to different capacitors through a rectifier in order to explore its capacitor charging performance. Figure 15a shows the PVEH-3 floater’s charging capacitors (10, 33, 47, and 100 µF) through a bridge rectifier circuit. At a vibration frequency of 2.7 Hz, ***a*** = 0.03 g, *H* = 23 mm, and *L* = 2 mm, after charging the capacitors with the device for 60 s, the voltage values of each capacitor reached 5.52, 5.24, 5, and 3 V. In addition, as shown in Figure 15b, a 33 µF capacitor was charged with a PVEH for 60 s, and the capacitor was connected to 333 parallel LED lamp electrodes. All of the 333 LED lamps were lit. Figure 16 shows the effect of a PVEH-3 charging a 33 µF capacitor for 60 s in different excitation directions. These results further demonstrate that the harvester can efficiently capture energy in ultra-low frequency, low-intensity, and 3D multidirectional vibration environments, and could have a wide range of application scenarios in the future.

Table 2 summarizes and compares several typically reported low-frequency multidirectional PVEHs with our PVEH in terms of the normalized power density, resonant frequency, and starting potential. As presented in Table 2, among the numerous PVEHs that harvest energy from the 2D space, the PVEH-R in this paper is considerably better than other energy harvesters in terms of the normalized energy density. In addition, compared with the PVEH-3 in this paper, the other PVEHs mentioned in the table that can harvest energy in 3D space encounter difficulty in harvesting vibrational energy under ultra-low-frequency (<3 Hz) and low-intensity (<0.04 g) excitation. Therefore, the proposed PVEH based on the rope-driven mechanism using liquid as a low-frequency energy-capture medium shows a broad application potential in low-frequency, ultra-low intensity, and multidirectional vibration energy harvesting in 3D space.

## 5. Conclusions

In this paper, an ultra-low frequency, low intensity, and multidirectional PVEH using liquid as the energy-capture medium was designed in view of the problems of a large starting potential and the low energy-capture efficiency of traditional vibration energy harvesters in low-frequency, low-intensity, and multidirectional environments. Based on the experimental results of the prototype, the device proposed in this article can achieve an output power of 51.55 µW under excitation at an ultra-low frequency (<3 Hz) and low intensity (<0.04 g), and the normalized power density can reach 45.94 μW/(cm^3^g^2^Hz). The observed intensity is notably lower than the intensity (>0.1 g) required for most PVEHs to achieve the same output power. Moreover, the prototype achieved 360° vibration energy harvesting in the horizontal plane and 120° in the vertical plane through one piezoelectric cantilever beam, which reduced the design difficulty of the energy management circuit. In addition, the resonant frequency of the device can be adjusted by changing the height of the liquid in the container, which improves the environmental adaptability of the device. The designed energy harvester is a multi-parameter coupling system, and the influence of each parameter on the output characteristics of the device was preliminarily studied in this paper. In future work, the coupling effect of different parameters on the output characteristics of the device can be further studied.

## Figures and Tables

**Table 1:** Parameters of the beam structure and piezoelectric elements (PZT 5J S230-J1FR-1808XB, Mide Technology Co., Woburn, MA, USA).

Parameter	Value
The length of the beam	71.1 mm
The width of the beam	25.4 mm
The thickness of beam	0.76 mm
Length of PZT 5J	46 mm
Width of PZT 5J	20.8 mm
Thickness of PZT 5J	0.15 mm
Resonant frequency	150 Hz
Spring constant	0.851 N/mm

**Table 2:** Performance comparison of the proposed PVEHs with the existing PVEHs.

References	Frequency (Hz)	Acceleration (g)	Excitations (2D/3D)	Power (μW)	Volume ^a^ (cm^3^)	Power Density (μW/(cm^3^g^2^Hz))
[30]	6.5	1.5	2D	180.00	8.47	1.45
[31]	17	0.4	2D	1000.92	55.8	6.59
[29]	5.2	2	2D	1228.8	19.2	3.08
[32]	16	0.5	3D	330.80	11	7.52
[26]	22	0.55	3D	280	137.3	0.31
This work	2.5	0.03	2D	48.71	471.24	45.94
This work	2.7	0.03	3D	20.56	471.24	17.96

^a^ Space demanded by the energy harvester during motions.

## Data Availability

Not applicable.
